# How a Gauze Sponge Roll Enhances Surgical Exposure in Thumb Carpometacarpal Arthroplasty: A Technical Note

**DOI:** 10.3390/jcm13206179

**Published:** 2024-10-17

**Authors:** Matthias Holzbauer, Julian Alexander Mihalic, Tobias Gotterbarm, Stefan Mathias Froschauer

**Affiliations:** 1 Department for Orthopedics and Traumatology, Kepler University Hospital GmbH, Krankenhausstrasse 9, 4020 Linz, Austria; julian.mihalic@kepleruniklinikum.at (J.A.M.); tobias.gotterbarm@kepleruniklinikum.at (T.G.); 2 Faculty of Medicine, Johannes Kepler University Linz, Altenberger Strasse 69, 4040 Linz, Austria; 3 Diakonissen Clinic Linz, Weißenwolffstraße 15, 4020 Linz, Austria; stefan.froschauer@maz.at

**Keywords:** gauze sponge roll, technical note, thumb carpometacarpal arthroplasty, trapeziometacarpal arthroplasty, touch prosthesis

## Abstract

Thumb carpometacarpal arthroplasty has become a widely used standard technique in the surgical treatment of thumb carpometacarpal osteoarthritis. One of the most critical steps during this procedure is proper surgical exposure of the trapezium and the base of the first metacarpal to allow for prosthesis implantation. This article introduces a surgical technique in which a roll constructed from gauze sponges tightly wrapped with medical tape facilitates several steps in thumb carpometacarpal arthroplasty. While performing a dorsoradial approach to the thumb carpometacarpal joint, this cost-effective tool is perfectly tailored to the joint’s unique anatomy. It aids in precise hand positioning and ensures optimal exposure of the trapezium and base of the first metacarpal, which is crucial for accurate cup and stem preparation as well as for unimpeded prosthesis implantation.

## 1. Introduction

Thumb carpometacarpal (CMC) arthroplasty has significantly evolved since its invention by de La Caffinière [1], with continuous advancements in implant design leading to the development of dual-mobility implants [2,3]. These innovations have effectively addressed the main issues faced by past generations, particularly low survival rates caused by aseptic cup loosening [2,3,4,5]. Recently, sustainable prosthesis fixation combined with satisfactory functional outcomes led many clinics and surgeons to include total joint arthroplasty (TJA) to their surgical repertoire for treating thumb CMC osteoarthritis (OA). While thumb CMC prosthesis was originally developed and for many years widely used in France [3], TJA has recently gained increasing popularity in German-speaking countries [6].

While thumb CMC TJA is emerging as standard treatment for thumb CMC OA, Prof. Hansen emphasizes in his review that “careful surgical technique is important to reduce the risk of implant failure” and thumb CMC TJA “remains a challenging surgery with a difficult learning curve” [3]. This observation is supported by several authors who report that technical errors are often the cause of their complications [7,8,9].

Given these challenges, this article aims to present our surgical technique, focusing on the use of a roll constructed from gauze sponges as a cost-effective tool to facilitate many crucial steps during thumb CMC TJA.

## 2. Surgical Technique

The tool facilitating thumb CMC TJA is crafted from five pieces of sterile, four-ply cotton gauze sponges, each measuring 20 × 30 cm, tightly wrapped with surgical tape. This forms a cylinder with a diameter of approximately 6 cm and a height of 10 cm.

For thumb CMC TJA using the Touch Prosthesis (KeriMedical, Les Acacias, Switzerland), we employ a dorsoradial approach centered in the anatomic snuffbox. The patient is positioned supine, and its forearm is placed on the hand table in neutral pro-/supination position.

**Figure 1:**
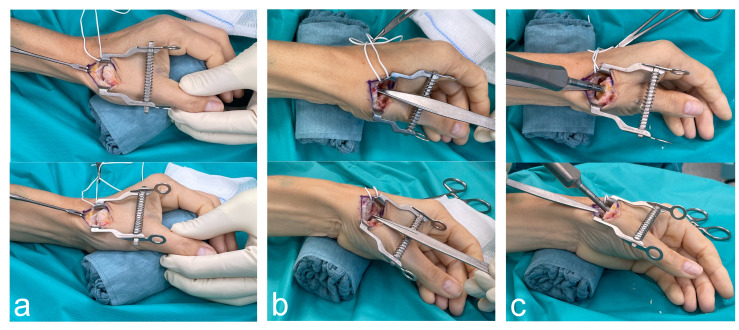
Intraoperative photographs displaying hand positioning on the gauze sponge roll during surgical approach to the thumb CMC joint (**a**); soft-tissue release, osteotomy and cup implantation (**b**); and stem implantation (**c**).

The first position of the gauze sponge roll is in the palm, supporting the thumb ray and simulating a pinch grip position, while the ulnar side of the hand lies on the surgical table (see Figure 1a).

The rationale for this position is that the contour of the gauze sponge roll provides support to the entire thumb ray while keeping the thumb CMC, the metacarpophalangeal, and the interphalangeal joints in relaxed position. This positioning enables the surgeon, seated beside the patient’s armpit, to have an optimal view of the surgical field during the procedure. Moreover, the mechanical support provided by the gauze sponge roll eliminates the need for the assistant surgeon to hold the thumb in place.

The procedure starts with a 2.5 to 3 cm long skin incision. Afterwards, the tendons of the first and third extensor compartments as well as the superficial branch of the radial nerve are identified. The radial artery is secured with a vessel loop. A self-retaining retractor is then inserted. The joint line of the thumb CMC joint capsule is identified and incised. Our technique includes resection of the entire dorsal joint capsule. Notably, Reischenböck et al. found no functional difference between patients who received a capsular resection or a capsular repair after 1 year of follow-up [10].

The second position of the gauze sponge role is under the ulnar aspect of the wrist with the forearm remaining in neutral pro-/supination (see Figure 1b,c). Anatomically, the sponge roll causes a maximal ulnar deviation in the wrist joint; hence, the scaphoid is maximally extended, which provided stability to the trapezium through the scaphotrapezial joint capsule [11].

As a next surgical step, a joystick-like technique is used to manipulate the first metacarpal. By radially and ulnarly deviating as well as rotating the first metacarpal, tension can be applied to each part of the thumb CMC capsule, facilitating a circumferential, sharp, and blunt soft-tissue release of the first metacarpal. Following, a Hohmann retractor is used to elevate and stabilize the first metacarpal for osteotomy. Osteotomy is performed perpendicular to the thumb with a slope to remove more bone palmarly to prevent impaction. The trapezium horns, whose prominence depends on the degree of the osteoarthritic degeneration, are resected with an oscillating saw. Care is taken not to resect the deepest point of the saddle-shaped surface because a circumferential cortical contact is crucial for the stability of the cup [12]. Any loose bodies or extensive synovitis are removed from the palmar joint capsule, which is clearly visible superficially in the flexor capi radialis tendon.

After ensuring adequate mobility of the first metacarpal, one or two small Hohmann retractors are employed to push the metacarpal palmarly, exposing the entire joint surface of the trapezium. This step is crucial for complete visualization of the exact margins of the trapezium’s joint surface so that the Kirschner wire can be precisely and unimpededly placed in its center (see Figure 1b). If the trapezium cannot be fully visualized from a distal viewpoint, additional soft-tissue release of the first metacarpal bone is required. This exposure, achieved by achieving maximal ulnar deviation in the wrist joint, facilitates the accurate positioning of the K-wire in the center of the trapezium’s proximal joint surface. The position of the K-wire is confirmed under fluoroscopy. The trapezial is reamed until the reamer is flush with the cortical bone. After the position and the angulation of the trial cup are checked under fluoroscopy, the prosthesis cup is implanted. The sponge roll ensures that these steps can be performed without interfering with the first metacarpal.

Next, the stem is implanted with the gauze sponge roll remaining in the same position. A Hohmann retractor is used to dorsally lift the base of the first metacarpal. Due to the extensive soft-tissue release, the Hohmann retractor uses the dorsal aspect of the trapezium as a lever while leaving the already implanted cup untouched and not jeopardizing its stability. While the surgical assistant holds the thumb in a flexed position, the medullary cavity is accessed at the transition of the dorsal to the middle third. Additional rasping is performed at a steep angle, preventing interference with the radius or the trapezium. Aligning the handle of the rasp with the thumb’s axis in two planes and respecting the aforementioned entry point assists in implanting the stem in the correct axis (see Figure 1c). The axis and dimensions of the rasp, which lead to rotational stability, are controlled under fluoroscopy. In this regard, some authors recommend avoiding entire cortical contact to prevent stress shielding [13]. The trial stem and the trail neck, typically with 15° angulation, are implanted. The stability of the prosthesis is checked via axial telescoping, aiming for subluxation of between half and full head length. Stability is also assessed while performing maximal opposition to the Kapandji position 10 [14]. After the final stem and neck are implanted, the gauze sponge role can be returned to its initial position for wound irrigation and skin closure.

## 3. Discussion

For thumb CMC TJA, this surgical technique introduces a cost-effective, handy tool: a roll constructed by surgical gauze sponges tightly wrapped with surgical tape. While several surgeons use folded large surgical towels as support underneath the wrist joint, the use of this sponge roll has not yet been described in the literature, to the best of our knowledge.

Maes–Clavier observed that mastering the technical procedure of thumb CMC TJA typically requires 30 procedures, highlighting the challenging learning curve of this procedure [7]. Moreover, these initial cases significantly influence the surgeon’s decision to adopt this procedure, especially if surgically more convenient options, such as trapeziectomy or resection–suspension–arthroplasties are available. Therefore, this technical note might prevent one of the following complications that are described to occur early in the experience of thumb CMC TJA due to enhanced surgical exposure.

Dumartinet reported that major technical errors, which led to the removal of 21 Arpe prostheses within the first year, included difficulties with reaming, incorrect implant positioning, and inadequate neck choice [8]. In our previous study, we observed two cases of EPL tendon rupture, potentially caused by sharp edges of the first metacarpal after osteotomy of the first metacarpal [9]. Moreover, Hansen notes that cup implantation requires “precise instrumentation and positioning in order to avoid excessive removal of bony circumferential support, loss of initial pressfit fixation, and fracture of the trapezium, which can lead to failure of osseointegration or even intra-operative trapeziectomy” [3]. Biomechanical studies confirm that the cup positioning is the most critical factor for stable mechanical fixation [12,15]. Surgical exposure is crucial during resection of the trapezium horns. It is important to avoid excessive resection of bone stock, as mechanical fixation is more stable in cortical bone than cancellous bone [12]. Maintaining at least 8 mm of trapezial height is essential to prevent intraoperative fractures [16,17]. Additionally, the orientation of the K-wire is essential for the optimal cup placement, i.e., the entry point should be located centrally in the trapezium in both planes to ensure circumferential cortical contact as well as prevent fracture during impaction [18]. The cup plane should be aligned parallel to the proximal surface of the trapezium, respecting the center of motion in physiological CMC joints [18], and minimizing the risk of prosthesis dislocation in all directions [15]. To master these crucial steps of choosing the correct entry point and angulation of the K-wire, perfect visualization of the trapezial surface is essential.

The technique described facilitates optimal detection of the trapezium’s margins to locate its center. Preoperative radiographs should be critically reviewed for prominent osteophytes to identify the “true” physiological center of the trapezium. Positioning on the sponge roll achieves maximal ulnar deviation and pushing the first metacarapal palmarly; thus, a wide range of angulation for perfect K-wire placement is enabled.

Moreover, Hansen emphasizes that the subluxation of the CMC joint, which is present in several OA patients, necessitates an extensive soft-tissue release [3]. In this regard, our technique enables ideal exposure and maneuverability of the first metacarpal for an extensive soft-tissue release.

The dorsoradial approach is recommended by both the manufacturer and the developer team of the Touch prosthesis led by Bruno Lussiez [19,20]. Historically, this approach has prevailed over the lateral–palmar approach. Advocates of the lateral–palmar approach suggested that the dorsal capsule is crucial for the prosthesis stability and can only be preserved via this technique [2]. However, several authors have shifted to the dorsoradial approach due to its superior exposure of the trapezium for precise cup positioning [8,21]. The study by Reischenböck et al. comparing capsular resection and repair in the dorsoradial approach refutes the importance of the dorsal capsule for prosthesis stability [10]. Capsular resection demonstrated non-inferiority regarding dislocation rates and did not exhibit higher infection rates compared to capsular repair, which was assumed to serve as an additional barrier preventing infection. Therefore, capsular resection is recommended to facilitate extensive release of the first metacarpal, enhancing postoperative range of motion by mobilizing the contracted soft tissue responsible for the adduction deformity. In a further consequence, this provides improved surgical exposure for accurate prosthesis positioning as discussed above.

## 4. Conclusions

A gauze sponge role provides a highly cost-effective tool for enhancing various steps in thumb CMC TJA. This can potentially reduce surgical time and lower the risk of certain complications, including inadequate soft-tissue release, causing restricted range of motion, and cup mispositioning, which can result in loosening or dislocation. Therefore, we recommend this technique for the dorsoradial approach in thumb CMC TJA.

## Data Availability

Data are contained within the article..
